# Long-noncoding RNA MALAT1 sponges microRNA-92a-3p to inhibit doxorubicin-induced cardiac senescence by targeting ATG4a

**DOI:** 10.18632/aging.103136

**Published:** 2020-05-08

**Authors:** Wenzheng Xia, Hanbin Chen, Congying Xie, Meng Hou

**Affiliations:** 1 Department of Neurosurgery, First Affiliated Hospital, Wenzhou Medical University, Wenzhou, China; 2 Department of Neurosurgery, Xinhua Hospital Affiliated to Shanghai Jiaotong University School of Medicine, Shanghai, China; 3 Department of Radiation Oncology, First Affiliated Hospital, Wenzhou Medical University, Wenzhou, China

**Keywords:** exosome derived from mesenchymal stem cells, hypoxia, doxorubicin related cardiac senescence, LncRNA-MALAT1/miR-92a-3p/ATG4a signaling pathway, mitochondrial metabolism

## Abstract

The clinical application of doxorubicin (Dox) is limited due to its undesirable cardiotoxicity side effects. Cellular senescence plays an important role in Dox-induced cardiotoxicity. Exosomes derived from stem cells showed a therapeutic effect in Dox-induced cardiomyopathy (DIC). Hypoxia-preconditioned exosomes (exosome^Hypoxia^) display pro-metabolism and pro-survival abilities. Several long-noncoding RNAs/microRNAs act as competing endogenous RNAs (ceRNAs) modulating DIC. No study investigated whether exosome^Hypoxia^ could attenuate DIC through modulating ceRNAs.

Treatment of the human adipose–derived mesenchymal stem cells with hypoxia induced lncRNA-MALAT1 accumulation in the secreted exosomes. In addition, the lncRNA-MALAT1 was identified as an exosomal transfer RNA to repress miR-92a-3p expression. Silencing the lncRNA-MALAT1 in MSCs or miR-92a-3p overexpression in cardiomyocytes significantly impaired the rejuvenation induced by exosome^Hypoxia^. TargetScan and luciferase assay showed that miR-92a-3p targeted the ATG4a 3' untranslated region. Silencing ATG4a blocked the anti-senescent effect of exosome^Hypoxia^.

This study identified the lncRNA-MALAT1 that functioned as ceRNA binding to miR-92a-3p, leading to ATG4a activation, thus improving mitochondrial metabolism. LncRNA-MALAT1/miR-92a-3p/ATG4a partially mediates the cardioprotective roles of exosome^Hypoxia^ in Dox-induced cardiac damage. Exosome^Hypoxia^ may serve as a potential therapeutic target against DIC.

## INTRODUCTION

With the improvement in cancer-related outcomes, cardiovascular disease has become a leading cause of morbidity and mortality among cancer survivors. The primary cause of cancer-related cardiomyopathy is chemotherapy. Most cardiotoxic agents include anthracyclines, such as Dox [1]. A retrospective analysis of the clinical use of Dox in adults showed that the incidence of congestive heart failure increased from 3% to 5% at a dose of 400 mg - m^−2^ and from 18% to 48% at a dose of 700 mg - m^−2^. Moreover, cardiac toxicity caused by Dox has serious consequences, resulting in a poor prognosis and death for up to 61% of patients [2, 3]. It is generally believed that Dox-induced cardiac toxicity mainly involves metabolism disruption, leading to cellular senescence [4, 5]. Based on the aforementioned mechanism, studies on Dox-induced senescence have attracted wide attention in recent years.

Mesenchymal stem cells (MSCs) have been widely accepted as an effective therapeutic approach in cardiovascular disorders [6]. Following the transplantation of MSCs, only a limited number of cells can engraft and differentiate into heart cells or participate in cardiac restoration [7]. Therefore, recent studies have focused on MSCs-derived exosomes as a safer alternative approach to MSCs therapy [8]. Exosomes are cell-derived vesicles containing proteins, lipids, growth factors, microRNAs (miRs), and anti-inflammatory cytokines released through exocytosis [9, 10]. Recent studies suggest that exosomes derived from stem cells have a therapeutic effect in Dox-induced cardiotoxicity [11]. Previous reports have convincingly shown that the quality and therapeutic function of MSCs are impacted by culture conditions [12]. Several *in vitro* studies have demonstrated that the hypoxic preconditioning causes distinctive changes in stem cell characteristics and influences the secretion of cytokines and growth factors [13]. Hypoxia-preconditioned MSCs showed a better therapeutic effect on radiation-induced cardiac damage [14]. A previous study revealed that exosomes from Ad-MSCs culture supernatants under hypoxic conditions could increase vascular tube formation [15]. Therefore, this study aimed to establish that exosomes derived from hypoxia-preconditioned MSCs could enhance the therapeutic effect in the Dox-induced cardiac cellular injury.

Long-noncoding RNAs (lncRNAs), which are a subset of noncoding RNAs with more than 200 bases, have been indicated in cardiac repairing [16]. Intriguingly, lncRNAs have emerged as novel modulators in the therapeutic effect on Dox-induced cardiac senescence [17]. Metastasis-associated lung adenocarcinoma transcript 1 (MALAT1), also known as nuclear-enriched transcript 2, has been identified as a prognostic biomarker of lung cancer metastasis and has been linked with several other types of human tumors [18]. A previous report concluded that MALAT1 was a hypoxia-inducible factor (HIF) [19]. Exosomes rich in the lncRNA-MALAT1 derived from MSCs showed rejuvenation potential [20]. MiRs have been shown to regulate multiple processes in cardiac pathophysiology [21]. MiR-containing exosomes derived from hypoxia-preconditioned MSCs showed a better therapeutic effect in cardiac damage [22]. Meanwhile, the role of miR-92a-3p has been confirmed in destroying cardiac homeostasis through inhibiting cardiomyocyte metabolism and autophagy, targeting ATG4a [23]. Enhanced ATG4a, as an autophagy inducer, rejuvenates the heart during the ischemia/reperfusion process [24]. To further explore its potential molecular biological mechanism, bioinformatics analysis was used, revealing that miR-92a-3p bound to MALAT1. Based on the aforementioned information, it was speculated that the exosomal lncRNA-MALAT1 might promote rejuvenation against Dox-induced cardiac senescence by regulating the miR-92a-3p/ATG4a axis.

The present study aimed to investigate the role of the lncRNA-MALAT1 transferred by exosomes^Hypoxia^ derived from MSCs in resisting Dox-induced cardiac senescence. It also explored the underlying molecular mechanisms to determine whether exosomes modulating lncRNA-MALAT1/miR-92a-3p/ATG4a could suppress Dox-induced senescence.

## RESULTS

### Exosomes derived from MSCs pretreated with hypoxia had a better cardioprotective effect

**Figure 1:**
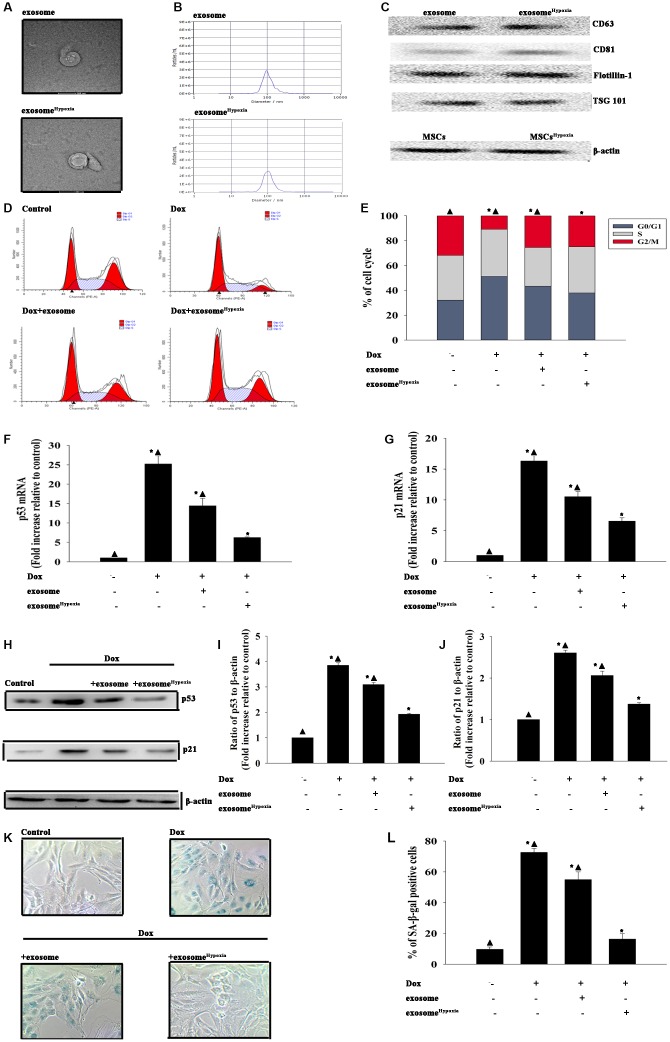
**Exosomes derived from MSCs pretreated with hypoxia had a better cardioprotective effect.** Confirmation of exosomal collection using TEM, NTA, and Western blot analysis. (**A**) Representative TEM image. (**B**) Size range of exosomes checked by NTA analysis. (**C**) Representative Western blot images showing that the exosomal marker CD63, CD81, Flotillin, and Tsg101 were highly expressed in exosome and exosome^Hypoxia^. β-actin in MSC lysate was examined. (**D** and **E**) Cell cycle distribution was analyzed. (**F** and **G**) p53 and p21 mRNA levels were analyzed using qRT-PCR. (**H**–**J**) p53 and p21 protein levels were analyzed using Western blot analysis. (**K**) Representative images of SA-β-gal staining. (**L**) Percentage of β-gal-positive cells. Each column represents the mean ± SD of three independent experiments. ^*^P < 0.05 versus control; ^▲^*P* < 0.05 versus Dox + exosome^Hypoxia^.

Considering the promoting effect of hypoxia preconditioning, the present study evaluated whether exosomes secreted by MSCs pretreated with hypoxia (exosome^hypoxia^) showed a more cardioprotective effect. First, the exosomes were isolated from the MSCs and MSCs treated with hypoxia as described earlier. Transmission electron microscopy (TEM) showed that exosomes exhibited a round-shaped morphology and a size of 50–100 nm. Moreover, the presence of the exosomal markers CD63, CD81, Flotillin-1, and Tsg101 was confirmed by Western blot analysis (Figure 1A–1C). No difference in concentration was reported between exosome and exosome^hypoxia^, confirmed by nanoparticle tracking analysis (NTA). Then, the effects of exosome^hypoxia^ on Dox-induced cardiomyocyte senescence were determined. The results revealed that compared with Dox-treated cardiomyocytes, exosome^hypoxia^ protected cardiomyocytes from senescence, showing that more cells escaped from the G0/G1 phase as measured using FACS (Figure 1D, 1E), with the decreased expression of the cellular senescence–related genes p53 and p21 (Figure 1F–1J) and a lower percentage of SA-β-gal-positive cells (Figure 1K, 1L). In the meanwhile, exosomes from MSCs without any treatment also elicited cellular rejuvenation to some extent, which was not significant compared with the exosome^Hypoxia^, indicating that hypoxia-preconditioned increased the cellular rejuvenation effect of exosomes.

### LncRNA-MALAT1 transferred by exosomes caused rejuvenation against Dox

**Figure 2:**
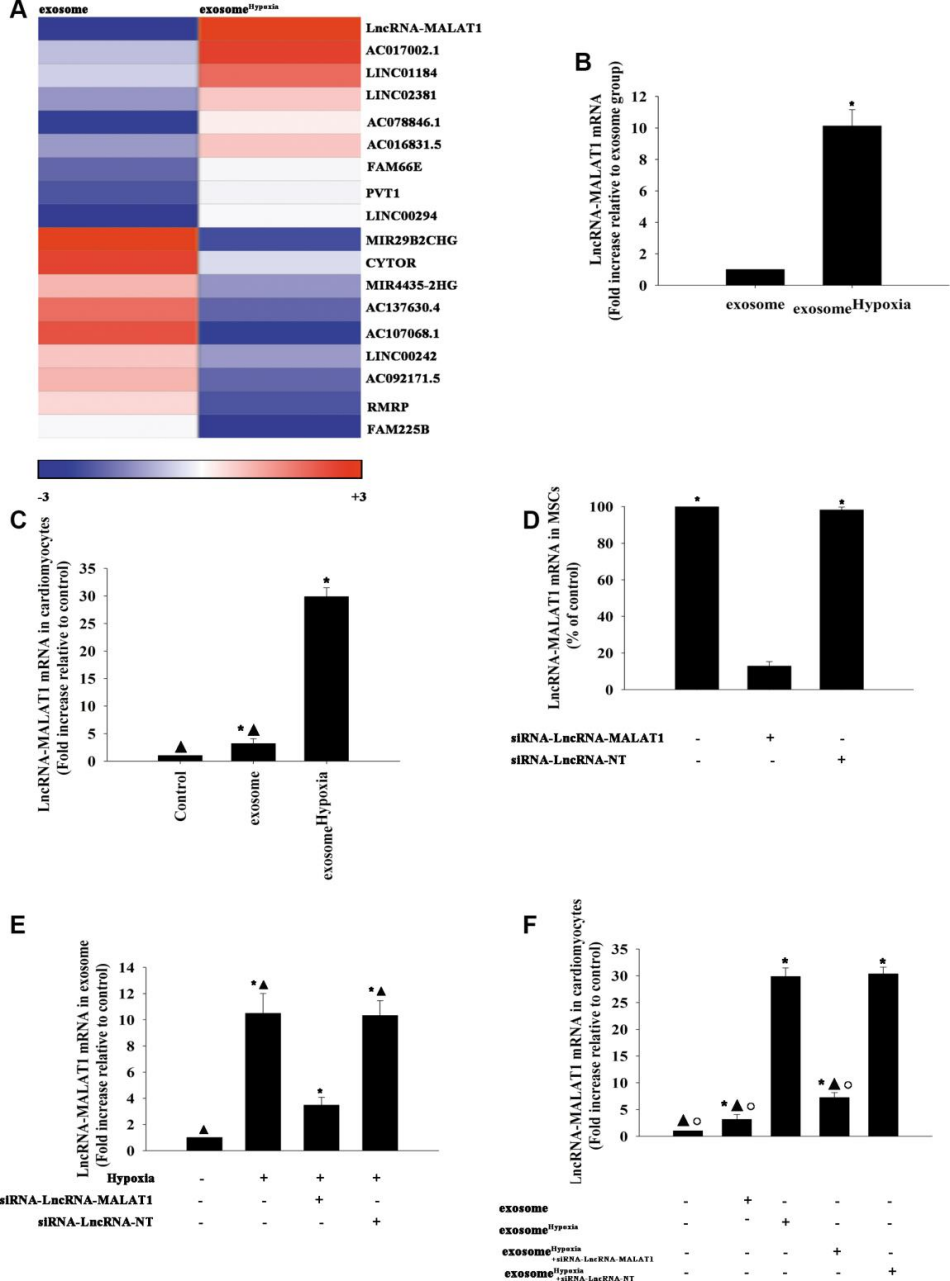
**LncRNA-MALAT1 transferred by exosomes to cardiomyocytes.** (**A**) Heat map of the lncRNAs differentially expressed between exosomes derived from MSCs pretreated with hypoxia (exosome^Hypoxia^) and exosomes derived from MSCs without any treatment (exosome). (**B**) Relative lncRNA-MALAT1 expression was validated by qRT-PCR in exosome^Hypoxia^ and exosome; ^*^P < 0.05 versus exosome; (**C**) LncRNA-MALAT1 mRNA was examined by qRT-PCR in cardiomyocytes incubated with exosome^Hypoxia^ or exosome. The cardiomyocytes without any treatment were used as control; ^*^P < 0.05 versus control; ^**▲**^*P* < 0.05 versus exosome^Hypoxia^. (**D**) The siRNA-mediated transfection efficiency in MSCs was demonstrated by qRT-PCR. ^*^P < 0.05 versus siRNA-lncRNA-MALAT1. (**E**) LncRNA-MALAT1 mRNA in exosomes was examined by qRT-PCR. Each column represents the mean ± SD of three independent experiments. ^*^P < 0.05 versus control; ^**▲**^*P* < 0.05 versus hypoxia + siRNA-lncRNA-MALAT1. (**F**) LncRNA-MALAT1 mRNA in cardiomyocytes was examined by qRT-PCR. ^*^P < 0.05 versus control; ^**▲**^*P* < 0.05 versus exosome^Hypoxia^; °*P* < 0.05 versus exosome^Hypoxia+siRNA-LncRNA-NT^

To assess the main biological pathways influenced by exosome^Hypoxia^, lncRNA analysis by microarray was conducted on MSC- and MSC^Hypoxia^ -derived exosomes. Genes significantly upregulated and downregulated in the exosome^Hypoxia^ group compared with the exosome group were identified. The maximum difference was for the lncRNA-MALAT1, a hypoxia-inducible lncRNA, which was confirmed by qRT-PCR (Figure 2A, 2B). To confirm whether this lncRNA could be transferred to the cardiomyocytes through exosomes, the lncRNA-MALAT1 mRNA was detected in the cardiomyocytes incubated with exosome^Hypoxia^ or exosome, compared with cardiomyocytes without any treatment. As expected, the lncRNA-MALAT1 showed the strongest upregulation in cardiomyocytes incubated with exosome^Hypoxia^, but also a little upregulation in the exosome group (Figure 2C). Furthermore, the expression of the lncRNA-MALAT1 in MSCs was silenced. The qRT-PCR results confirmed that the silencing of the lncRNA-MALAT1 significantly decreased the expression of the lncRNA-MALAT1 in MSCs (Figure 2D). MSCs were transfected with siRNA against the lncRNA-MALAT1 or control siRNA-NT and were cultured under hypoxic conditions. After, the exosomes were collected. The LncRNA-MALAT1 mRNA in exosomes was examined using qRT-PCR, revealing that hypoxia increased lncRNA-MALAT1 generation, which was abolished by lncRNA-MALAT1 silencing (Figure 2E). Exosomes derived from MSCs, which were transfected with siRNA against the lncRNA-MALAT1 or control siRNA-NT and treated with hypoxia or treated only with hypoxia, or without any treatment, were added to cardiomyocytes. In parallel experiments, cardiomyocytes without any treatment were used as control. Then, lncRNA- MALAT1 mRNA in cardiomyocytes was examined by qRT-PCR. The results revealed that exosome^Hypoxia^ transferred the lncRNA-MALAT1 to cardiomyocytes, while this transfer was blocked by endogenous lncRNA-MALAT1 silencing in MSCs (Figure 2F).

**Figure 3:**
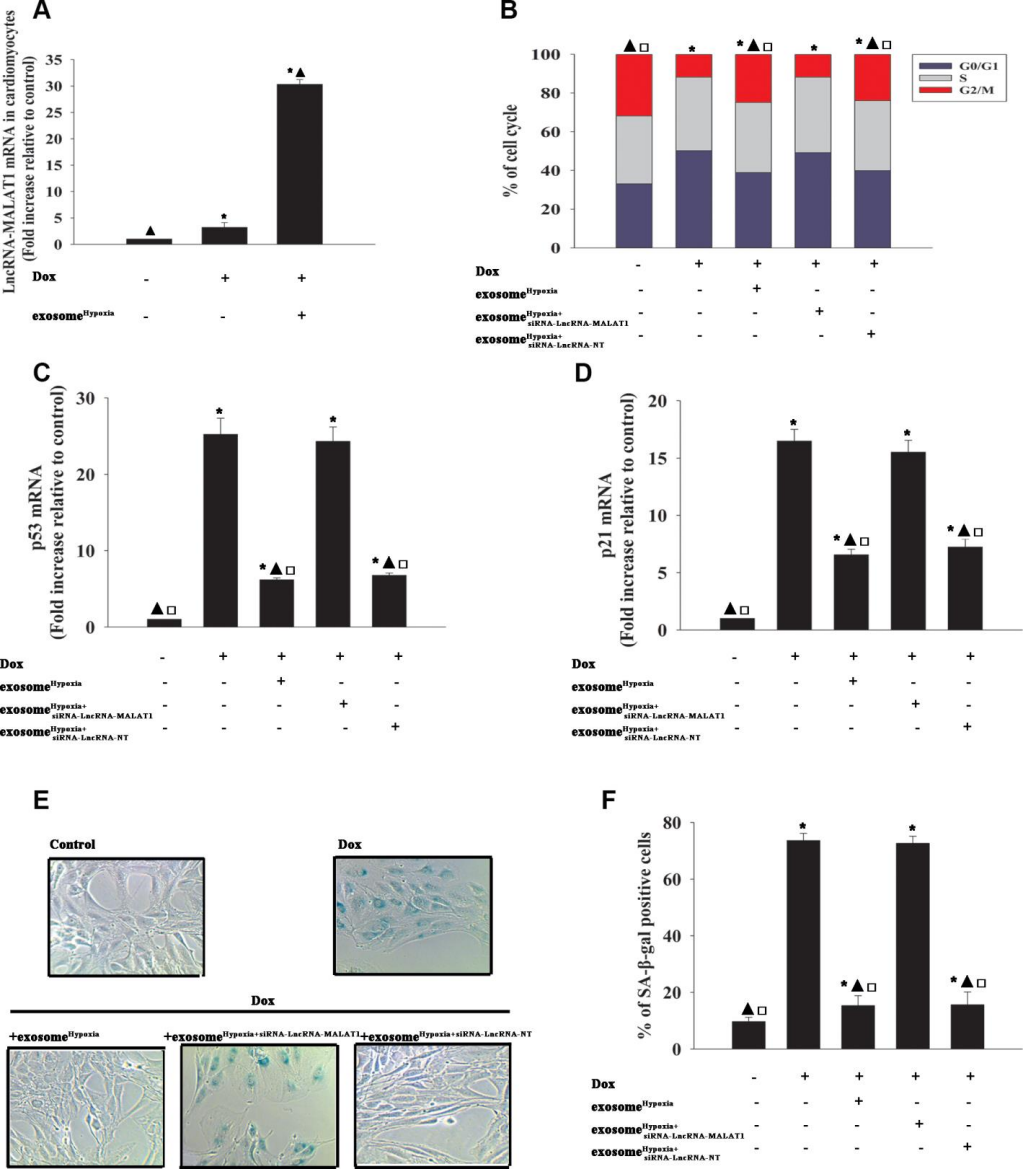
**LncRNA-MALAT1 transferred by exosomes caused rejuvenation against Dox.** (**A**) LncRNA-MALAT1 mRNA in cardiomyocytes was examined by qRT-PCR. ^*^P < 0.05 versus Control; ^**▲**^*P* < 0.05 versus Dox. (**B**) Cell cycle distribution was analyzed. (**C** and **D**) p53 and p21 mRNA levels were analyzed by RT-qPCR. (**E**) Representative images of SA-β-gal staining. (**F**) Percentage of β-gal-positive cells. Each column represents the mean ± SD of three independent experiments. ^*^P < 0.05 versus control; ^**▲**^*P* < 0.05 versus Dox; ^□^*P* < 0.05 versus Dox + exosome^Hypoxia+siRNA-lncRNA-MALAT1^.

In the subsequent experiments, exosomes derived from MSCs, which were transfected with siRNA against the lncRNA-MALAT1 or control siRNA-NT and treated with hypoxia or treated only with hypoxia, were added after cardiomyocytes were treated with Dox. In parallel experiments, cardiomyocytes without any treatment were used as control. As shown in Figure 3A, Dox alone increased the expression of the lncRNA-MALAT1 slightly. Dox induced cardiac senescence, while exosome^Hypoxia^ significantly reduced the percentage of G0/G1 phase cells (Figure 3B), expression of classical senescence-associated genes (Figure 3C and 3D), and number of SA-β-gal-positive cells (Figure 3E and 3F). This protective effect was abolished by silencing the lncRNA-MALAT1 in MSCs before treatment with hypoxia. These results strongly supported that exosomes derived from MSCs^Hypoxia^ exerted a rejuvenation effect partially through direct lncRNA-MALAT1 transfer.

### LncRNA-MALAT1 directly inhibited mir-92a-3p against Dox-induced senescence

**Figure 4:**
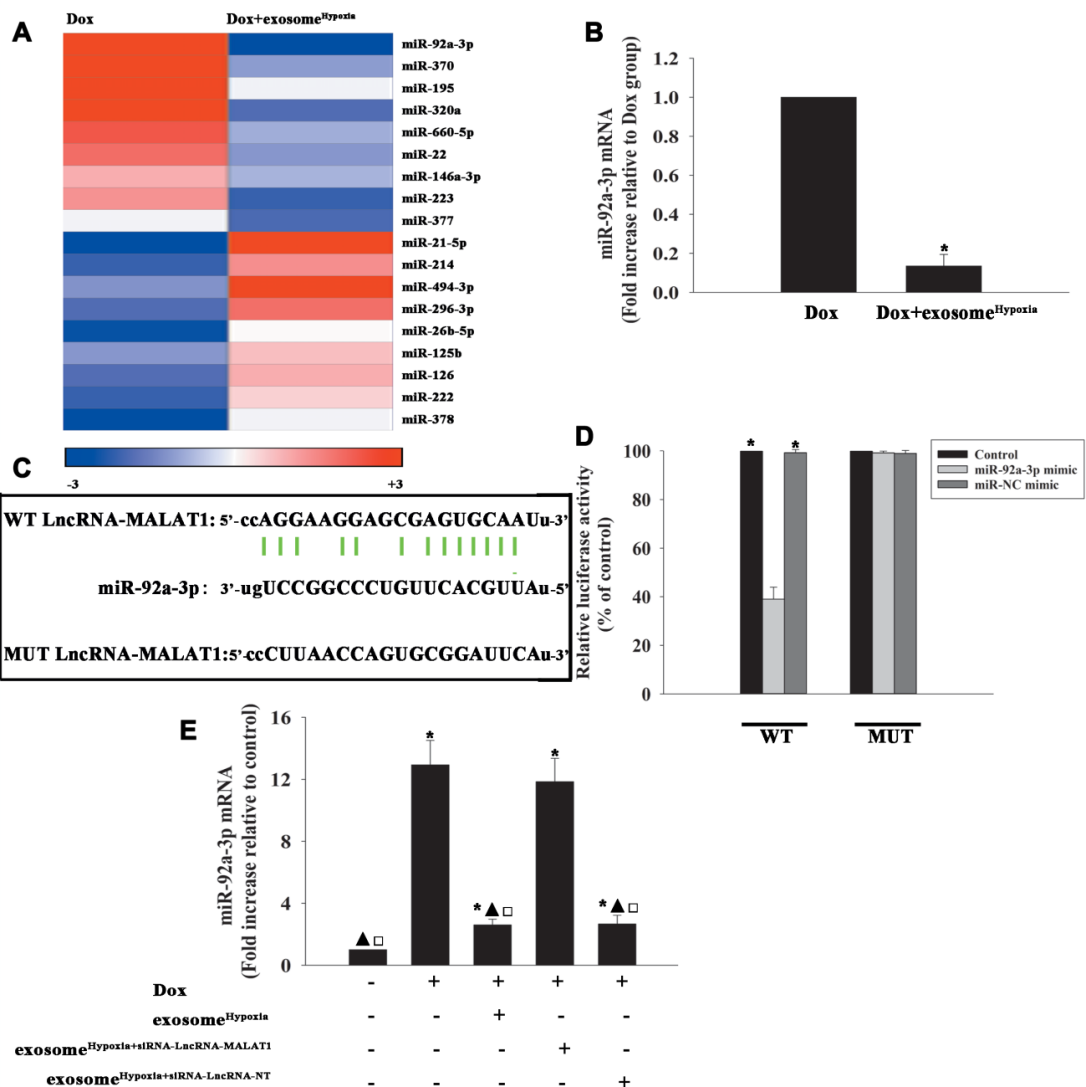
**LncRNA-MALAT1 directly inhibited miR-92a-3p.** (**A**) Heat map of microRNAs (miRs) differentially regulated by exosome^Hypoxia^ in Dox-treated cardiomyocytes. (**B**) qRT-PCR validation of the differentially regulated miRs in cardiomyocytes. ^*^P < 0.05 versus Dox. (**C**) Binding sites of lncRNAs and miRs. (**D**) Dual-luciferase reporter. ^*^P < 0.05 versus miR-92a-3p mimic in the WT group. (**E**) miR-92a-3p mRNA levels were analyzed by RT-qPCR. ^*^P < 0.05 versus control; ^▲^*P* < 0.05 versus Dox; ^**□**^*P*<0.05 versus Dox + exosome^Hypoxia+siRNA-lncRNA-MALAT1^.

To investigate the functional molecules responsible for the rejuvenating effect of exosome^Hypoxia^, the miR expression levels of Dox-treated cardiomyocytes and exosome^Hypoxia^ added into Dox-treated cardiomyocytes were analyzed using a microarray (Figure 4A). High levels of miR-92a-3p were detected in the Dox model compared with the Dox+ exosome^Hypoxia^ group, which was confirmed by qRT-PCR (Figure 4B). Also, the bioinformatics database (Lncbase) was analyzed. MiR-92a-3p was identified as being capable of binding to complementary sequences in the lncRNA-MALAT1 (Figure 4C). The luciferase reporter assay was used to prove the prediction. The results showed that the lncRNA-MALAT1-wild type (WT) could significantly lower the luciferase activity in the miR-92a-3p mimic group, but had no significant effect on the luciferase activity in the miR-negative control (NC) mimic group (Figure 4D). To determine whether the expression of miR-92a-3p was downregulated by the lncRNA-MALAT1 induced by exosome^Hypoxia^, MSCs were transfected with the siRNA-lncRNA-MALAT1 or siRNA-lncRNA-NT and then subjected to hypoxia; the exosomes were collected. Dox-treated cardiomyocytes expressed a higher level of miR-92a-3p. However, the expression level of miR-92a-3p in Dox-treated cardiomyocytes substantially reduced when exosome^Hypoxia^ was added. Exosomes derived from hypoxia-preconditioned MSCs with silenced lncRNA-MALAT1 could not alleviate the Dox-induced increase in the expression level of miR-92a-3p (Figure 4E).

**Figure 5:**
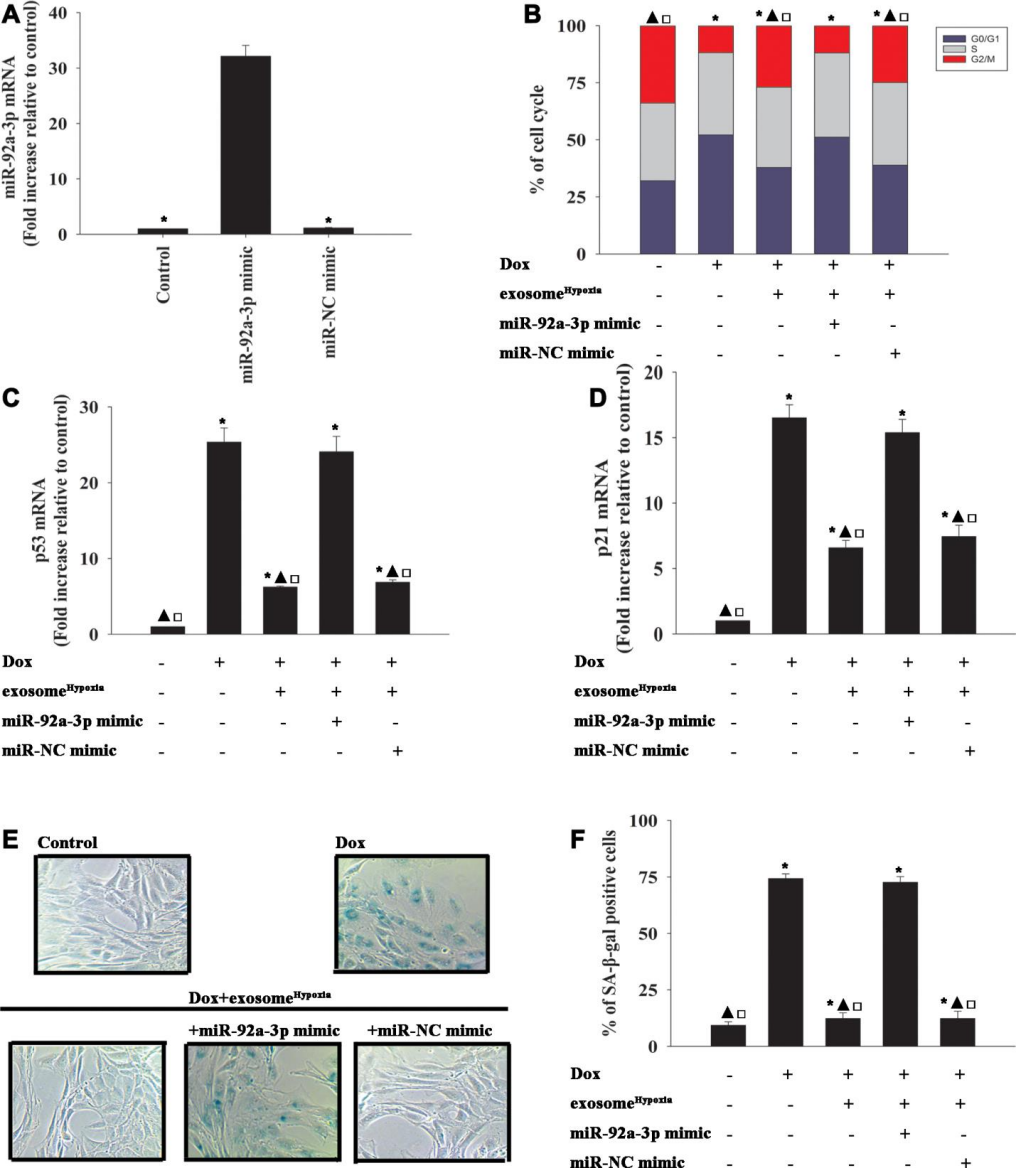
**miR-92a-3p impeded exosome^Hypoxia^ anti-senescent effect against Dox.** (**A**) miR-92a-3p mRNA levels were analyzed by qRT-PCR. ^*^P < 0.05 versus miR-92a mimic. (**B**) Cell cycle distribution was analyzed. (**C** and **D**) p53 and p21 mRNA levels were analyzed by qRT-PCR. (**E**) Representative images of the SA-β-gal staining. (**F**) Percentage of β-gal-positive cells. Each column represents the mean ± SD of three independent experiments. ^*^P < 0.05 versus control; ^▲^*P* < 0.05 versus Dox; ^**□**^*P* < 0.05 versus Dox + exosome^**Hypoxia**^ + miR-92a-3p mimic.

Considering the inhibitory action of the lncRNA-MALAT1 on the expression levels of miR-92a-3p in cardiomyocytes, the role of miR-92a-3p in the rejuvenating effects of exosome^Hypoxia^ against Dox-induced senescence was further explored. Cardiomyocytes were transfected with a mimic control or the miR-92-3p mimic (Figure 5A) and treated with exosome^Hypoxia^ and Dox. In parallel, cardiomyocytes treated or untreated with the exosome^Hypoxia^ were treated with Dox. The untreated cardiomyocytes were used as control. Dox treatment increased the percentage of G0/G1 phase cells (Figure 5B), expression of classical senescence-associated genes p53 and p21 (Figure 5C, 5D), and the number of SA-β-gal-positive cells (Figure 5E, 5F), indicating that Dox treatment induced cellular senescence. Exosome^Hypoxia^ alleviated Dox-induced cellular senescence, while transfection with an miR-92a-3p mimic further aggravated cell damage (Figure 5B–5F).

### Exosomal lncRNA-MALAT1/miR-92a-3p activated ATG4a to cause rejuvenation

**Figure 6:**
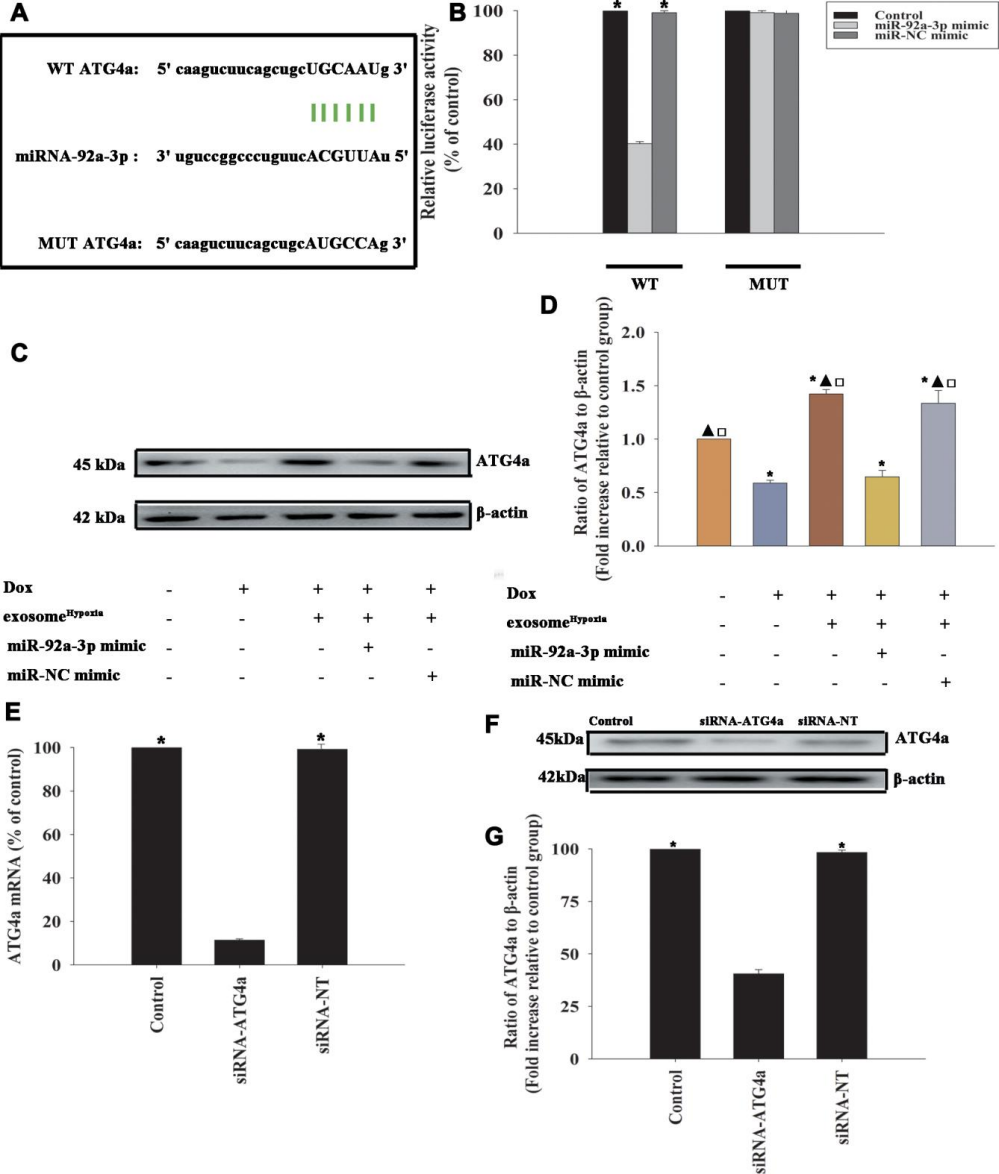
**ATG4a was a direct target of miR-92a-3p.** (**A**) The predicted binding sites between miR-92a-3p and the ATG4a 3'-UTR. (**B**) A dual-luciferase assay was performed in cardiomyocytes after co-transfection with ATG4a 3'-UTR wild type (WT) or mutant (MUT) plasmids, miR-92a-3p mimic, and miR-NC mimic. ^*^P < 0.05 versus the miR-92a-3p mimic in the WT group. (**C** and **D**) Western blot analysis of ATG4a and β-actin protein levels in cardiomyocytes. Untreated cardiomyocytes were used as control. ^*^P < 0.05, versus Control; ^▲^*P* < 0.05 versus Dox; ^**□**^*P* < 0.05 versus Dox + exosome^**Hypoxia**^ + miR-92a-3p mimic. (**E**–**G**) Cardiomyocytes were transfected with siRNA-ATG4a or with siRNA-NT as control. The siRNA-mediated transfection efficiency was determined by qRT-PCR (**E**) and Western blot analysis (**F** and **G**). Each column represents the mean ± SD from three independent experiments. ^*^P < 0.05 versus siRNA-ATG4a.

To identify the target genes of miR-92a-3p regulation, the bioinformatics database was used to speculate a putative binding site between miR-92a-3p and ATG-4a (Figure 6A), which was confirmed by dual-luciferase gene reporter assay. The relative luciferase activity was significantly weakened in the WT ATG4a + miR-92a-3p mimic group (Figure 6B). To investigate the inhibition effect of miR-92a-3p on ATG4a expression, the cardiomyocytes were transfected with an miR-92a-3p mimic or an miR-NC mimic, treated with exosome^Hypoxia^, and then subjected to Dox; the untreated cardiomyocytes were used as control. As expected, Dox treatment markedly inhibited ATG4a expression in cardiomyocytes, while exosome^Hypoxia^ rescued the expression of ATG4a. In addition, extra miR-92a-3p impaired the expression of ATG4a (Figure 6C, 6D). These data suggested that ATG4a were genuine targets of miR-92a-3p.

**Figure 7:**
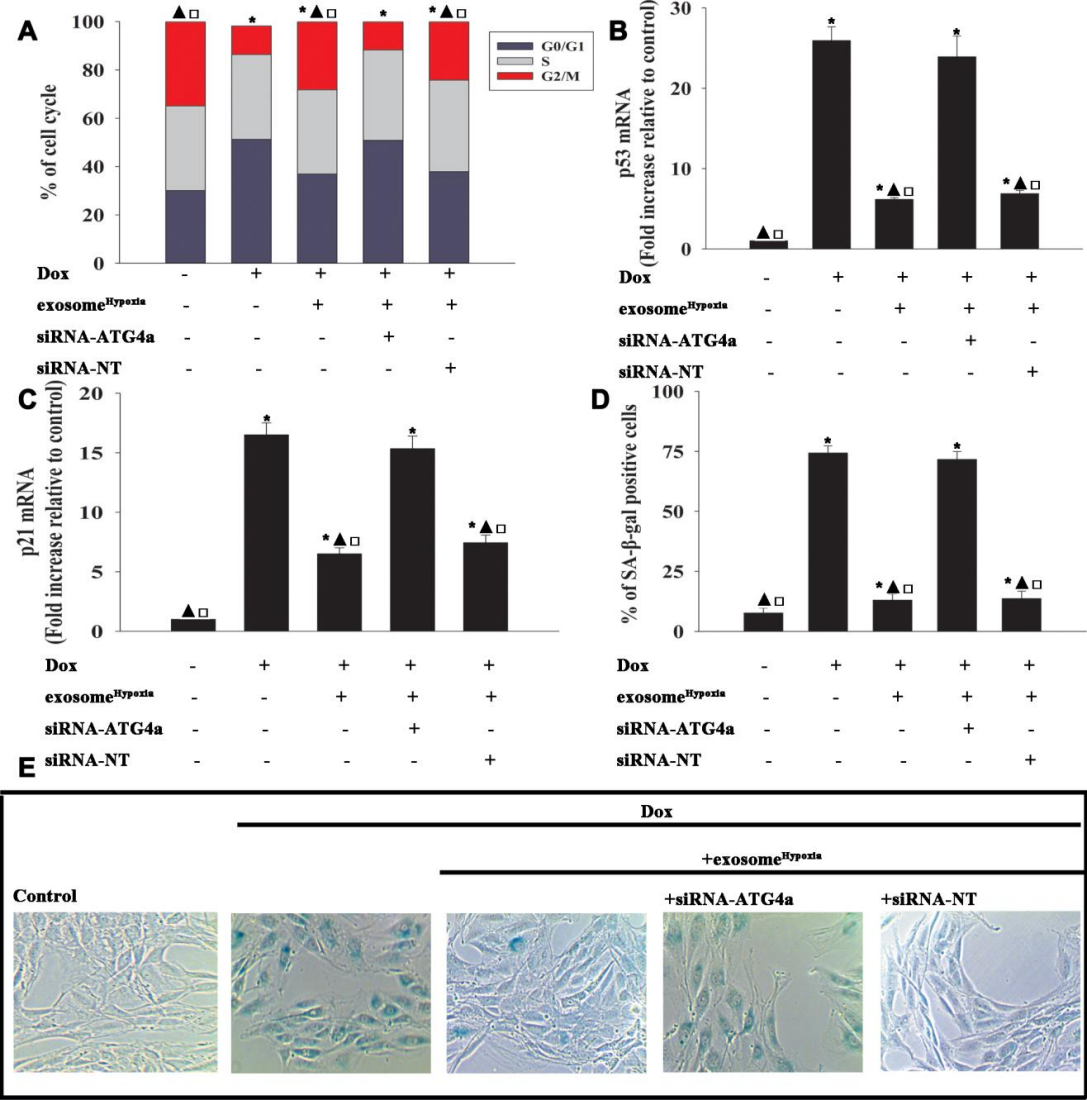
**Exosomal lncRNA-MALAT1/miR-92a-3p activated ATG4a to cause rejuvenation.** (**A**) Cell cycle distribution was analyzed. (**B** and **C**) p53 and p21 mRNA levels were analyzed by qRT-PCR. (**D**) Percentage of β-gal-positive cells. (**E**) Representative images of SA-β -gal staining. Each column represents the mean ± SD of three independent experiments. ^*^P < 0.05 versus control; ^▲^*P*<0.05 versus Dox; ^**□**^*P* < 0.05 versus Dox + exosome^**Hypoxia**^ + siRNA-ATG4a.

To explore the role of ATG4a in exosome^Hypoxia^-induced rejuvenation against Dox, ATG4a in cardiomyocytes was silenced by siRNA, and this caused a reduction in the expression of ATG4a, confirmed by qRT-PCR (Figure 6E) and Western blot analysis (Figure 6F, 6G). The cardiomyocytes were transfected with siRNA-ATG4a or siRNA-NT as control, treated with exosome^Hypoxia^, and subjected to Dox. In parallel, cardiomyocytes treated or untreated with exosome^Hypoxia^ were subjected to Dox. The untreated cardiomyocytes were used as control. ATG4a knockdown restored the pro-senescent effects of Dox inhibited by exosome^Hypoxia^ and increased the percentage of G0/G1 phase cells (Figure 7A), expression of classical senescence-associated genes p53 and p21 (Figure 7B, 7C), and number of SA-β-gal-positive cells (Figure 7D, 7E).

### Exosome^Hypoxia^ modified mitochondrial metabolism of cardiomyocytes disturbed by Dox

**Figure 8:**
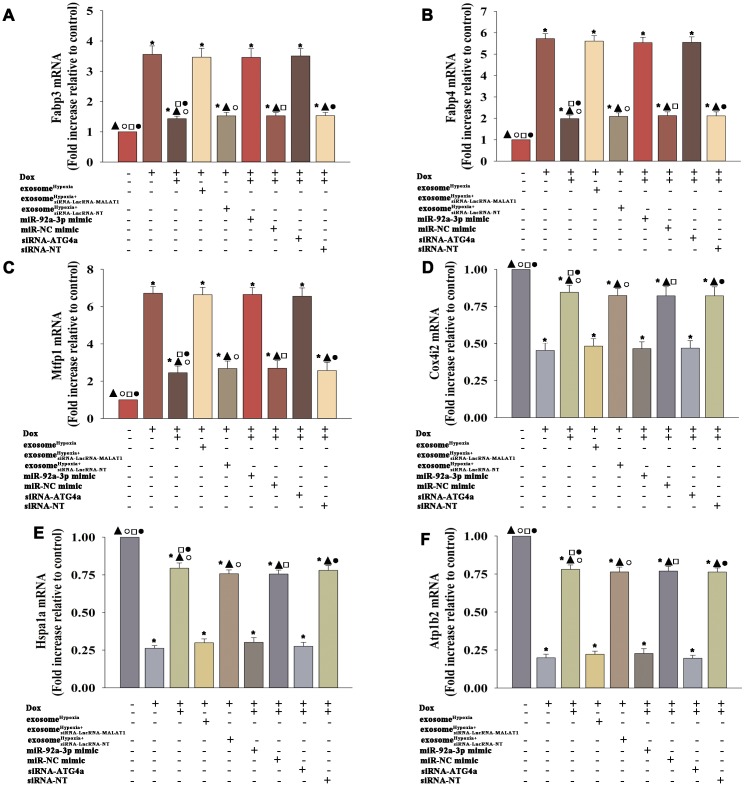
**Exosome^Hypoxia^ modified the metabolism of cardiomyocytes disturbed by Dox.** (**A**) mRNA level of Fabp3, (**B**) mRNA level of Fabp4, (**C**) mRNA level of Mtfp1, (**D**) mRNA level of Cox4i2, (**E**) mRNA level of Hspa1a, and (**F**) mRNA level of Atp1b2. ^*^P < 0.05 versus Control; ^▲^*P* < 0.05 versus Dox; °*P* < 0.05 versus Dox + exosome^Hypoxia+siRNA-LncRNA-MALAT1^; ^□^*P* < 0.05 versus Dox + exosome^Hypoxia^ + miR-92a-3p mimic; ^●^*P* < 0.05 versus Dox + exosome^Hypoxia^ +siRNA-ATG4a.

Cardiomyocytes showed a profound metabolism disruption during the senescence process [25]. Therefore, this study aimed to gain insight into the improvement of exosome^Hypoxia^ in mitochondrial metabolism. MSCs were transfected with siRNA against the lncRNA-MALAT1 or control siRNA-lncRNA-NT and then treated with hypoxia. The exosomes were collected and added to cardiomyocytes treated with Dox. The cardiomyocytes were transfected with miR-92a-3p mimic, miR-NC mimic, siRNA-ATG4a, or siRNA-NT; treated with exosome^Hypoxia^; and subjected to Dox. Cardiomyocytes, with or without exosome^Hypoxia^, were subjected to Dox. The untreated cardiomyocytes were used as control. The expression levels of fatty acid–binding proteins 3 and 4 (Fabp3 and Fabp4) (Figure 8A, 8B) and mitochondrial fission process 1 (Mtfp1) protein (Figure 8C) increased, while the expression levels of cytochrome c oxidase subunit 4I2 (Cox4i2) (Figure 8D), heat shock protein family A member 1A (Hspa1a) (Figure 8E), and ATPase Na+/K+ transporting subunit beta 2 (Atp1b2) (Figure 8F) decreased in cardiomyocytes treated with Dox. A significant reverse effect was noted when exosome^Hypoxia^ was added. The expression levels of Fabp3, Fabp4, and Mtfp1 increased and the expression levels of Cox4i2, Hspa1a, and Atp1b2 decreased after lncRNA-MALAT1 knockdown, corresponding miR-92a-3p overexpression, or silencing of ATG4a (Figure 8A–8F), suggesting that the exosome^Hypoxia^-induced lncRNA-MALAT1/miR-92a-3p/ATG4a signaling pathway improved mitochondrial metabolism in Dox-treated cardiomyocytes.

## DISCUSSION

Cardiovascular diseases and cancer represent the first and second most causes of death in industrialized countries, respectively. These two conditions may become synergistic if the cardiovascular complications of anticancer therapies are considered [26]. Cardiac dysfunction triggered by Dox has long been known as the main form of anticancer drug–induced cardiotoxicity. It is characterized by metabolism disruption and related cellular senescence as central mechanisms [27, 28]. Metabolism disorders and senescence consist of the growth arrest of normal somatic and postmitotic cells with a consequent reduction in anthracycline-driven cardiotoxic effects, injured the functional activity of cardiomyocytes and other cardiac cells, thus leading to functional and organ damage [29]. The present study demonstrated that Dox treatment enhanced cellular senescence in cardiomyocytes. The data further revealed that Dox-induced senescence was characteristic of cell cycle arrest. Cardiac cells responded to Dox, accompanied by metabolism disruption [30]. Many studies have shown that ameliorating metabolism before administering Dox protects against Dox toxicity [31, 32]. Amelioration of metabolism in the early stage of Dox-induced myocardial toxicity, and thus relief from cardiac senescence, might help in the treatment of DIC.

Stem cells have been widely used to treat various animal and human cardiac disorders [33, 34]. Stem cell–derived cardiomyocytes have been shown to repair Dox-induced heart failure [35, 36]. However, published reports showed potential chances of tumor formation and low survival rate after transplantation [37, 38]. A recent discovery of a cell-free system using exosomes has generated a new era in stem cell therapy for cardiac repair and regeneration [39, 40]. A recent report suggested that exosomes derived from stem cells could reduce Dox-induced adverse cardiac remodeling, including cytoplasmic vacuolization, myofibril loss, cardiac hypertrophy, and fibrosis [11]. Several strategies have been developed to improve the performance of MSCs for therapeutic applications. For example, Akt-modified MSCs could repair infarcted myocardium [41]. Hypoxic preconditioning of MSCs has proven to be a useful approach to increase their therapeutic potential in a model of hindlimb ischemia [42]. Exosomes derived from HIF-1α-overexpressing MSCs increased their therapeutic potential by potentiating angiogenesis [39]. This study further evaluated whether these exosomes could be optimized to exert an effect on Dox-induced cardiac dysfunction. The intercellular transfer of exosomes is a well-established messenger system that mediates cell–cell communication [43]. The information transfer induced by exosomes is time and environment-specific [44]. The exosomes derived from hypoxia-preconditioned MSCs consist of growth factors important in resisting cellular senescence [15]. Therefore, this study aimed to characterize exosomes derived from MSCs under hypoxic conditions. It focused on the enhanced rejuvenation ability of exosome^Hypoxia^ against Dox-induced cardiac senescence. Moreover, recovery from cell cycle arrest and the decrease in the expression levels of senescence–related genes and percentage of SA-β-gal-positive cells supported the notion that exosome^Hypoxia^ showed better therapeutic potential in Dox-induced cardiac senescence.

Accumulating evidence has shown that lncRNAs participate in a variety of biological processes. Meanwhile, the lncRNA sequencing analysis reveals that the expression of lncRNAs is, in general, species-specific and participates in cardiac regeneration [45]. For example, lncRNA-CAREL in the human genome is capable of regulating the proliferation of human iPS-derived cardiomyocytes, showing significant potential in cardiac regeneration after injury [46]. The aging process is a major risk factor for cardiovascular diseases. Further, Dox-induced cellular senescence caused cardiovascular dysfunction [47]. A previous study found that modulating the expression of lncRNA in cardiomyocytes relieved Dox-induced senescence [17]. The present study showed that the beneficial effects of exosomes were mediated by lncRNA MALAT1 transferring in the treatment of Dox-induced cardiac senescence. The loss-of-function approach by silencing lncRNA MALAT1 in MSCs demonstrated that the function of exosomes was mediated by the lncRNA MALAT1 pathway. This finding not only supported the previous reports that lncRNA MALAT1 was a hypoxia-inducible factor that regulated cell cycle and took a protective role in cardiac damage [48, 49], but also demonstrated that exosomes could be used to deliver lncRNA MALAT1 to alter Dox-induced senescence, which might have clinical implications.

A previous study suggested that lncRNAs might act as ceRNAs to interact with miRs and influence the expression of miR target genes [50]. The lncRNA H19 has been shown to bind to miR-103/107 and inhibiting FADD expression and necrosis in a mouse model of ischemia/reperfusion [51]. APF as a ceRNA for miR-188-3p suppresses autophagic cell death and cardiac injuries by targeting ATG7 [45]. Also, CAREL was predicted to have the potential to bind to miR-296-3p and induce cardiac regeneration [46]. This study further investigated the potential targets of the lncRNA-MALAT1 using bioinformatics analysis, and miR-92a-3p was selected. Previous studies demonstrated that the inhibition of miR-92a-3p in the ischemia/reperfusion experiments could decrease cardiomyocyte apoptosis and exert a protective effect [52]. The finding that mitochondrial metabolism increased after miR-92a-3p depletion in myocardial infarction offered interesting and new insights that miR-92a-3p depletion treatment might exhibit cardioprotective effects by improving the metabolism in cardiomyocytes [23]. *In vitro* experiments were conducted to investigate a putative direct effect of miR-92a-3p on Dox-induced cardiomyocyte senescence. In this *in vitro* model, the miR-92a-3p overexpression abolished the anti-senescent effect of exosome^Hypoxia^ against Dox. Next, ATG4a was established as the target gene of miR-92a-3p. and the ATG4a is an autophagic gene regulating the formation of autophagosomes [53]. Consistently, previous studies also reported that the overexpression of ATG4a resulted in considerable rejuvenation through modulating autophagy [54]. When autophagy was stimulated by an inducer, a double-membrane phagophore proximal to the cellular cargos expanded to generate autophagosomes, which ultimately fused with the late endosome or lysosome to hydrolyze the engulfed constituents, thus revealing Dox-induced cardiotoxicity [55]. As an important protein in autophagy, ATG4a was inhibited by Dox, while exosome^Hypoxia^ rescued ATG4a. However, the inducement of ATG4a by exosome^Hypoxia^ was hampered by miR-92a-3p overexpression. This study demonstrated that exosome^Hypoxia^-derived lncRNA-MALAT1 inhibited miR-92a-3p, causing activation of ATG4a against Dox-induced cardiac damage.

The cardiotoxicity of Dox is generally attributed to mitochondrial damage and induction of cell senescence in cardiomyocytes [5, 56]. The mitochondrion plays a central role in energy production and cellular respiration, as well as in cell senescence [57]. More than 90% of the adenosine triphosphate (ATP) used by cardiomyocytes is produced by mitochondria mainly through fatty acid utilization [58]. Mitochondrial respiration provides basal energy required for the normal metabolism of cardiomyocytes and holds in reserve the potential for maximal respiration if required. This potential, the so-called spare respiratory capacity, serves the increased energy demand for maintaining cellular functions under stress [59]. Dox can disrupt the electron transport chain within the mitochondrion and induce multiple forms of cellular injury to cardiomyocytes, including metabolism disturbance and cellular senescence [60, 61]. Coincident with previous findings, exosome^Hypoxia^ decreased the expression of metabolism-related genes, such as Fabp3 and Fabp4, which impaired cardiac metabolism and aggravated cardiac dysfunction in cardiac injury [62, 63]. Exosome^Hypoxia^ also downregulated the expression levels of Mtfp1, a participant in Dox-induced cardiac injury [64]. While, exosome^Hypoxia^ increased the expression levels of Hspa1a (autophagy inducer) [65], Cox4i2 (the modulator of mitochondrial response) [66], and ATP1B2 (cardiac oxidative stress inhibitor) [67], accompanied by cellular rejuvenation. Also, these metabolism-related genes were the target genes of miR-92a-3p to maintain cardiac metabolism homeostasis [23].

In summary, the results showed that exosomes derived from hypoxia-treated MSCs markedly enhanced rejuvenation in the Dox-treated cardiomyocytes. The associated mechanism was also deciphered. Specifically, exosomes transferred the lncRNA-MALAT1, inhibiting miR-92a-3p, leading to activation of ATG4a, thereby improving mitochondrial metabolism, and synergistically promoting rejuvenation. Therefore, hypoxic preconditioning of MSCs-derived exosomes might represent a novel strategy for the clinical treatment of DIC. However, the present study lacked *in vivo* data, and hence further studies are needed.

## MATERIALS AND METHODS

### Cell culture and treatment

### Human-induced pluripotent stem cell–derived cardiomyocytes

Human-induced pluripotent stem cell–derived cardiomyocytes were obtained from Cellular Dynamics International (WI, USA) and plated and maintained according to the manufacturer’s guidelines. The cells were plated on 10 μg/mL fibronectin medium (Invitrogen 33016-015, CA, USA) as a support matrix for 48 h at 37°C and 5% CO_2_. The maintenance medium was changed every 2 days.

### Human adipose–derived MSCs

Human Ad-MSCs, purchased from ATCC, were cultured in an early-passage DMEM–F12 medium, according to the supplier’s specifications, and incubated at 37°C and 5% CO_2_. The medium was changed every 48 h. The cells were harvested using 0.05% Trypsin-EDTA (cat. number 25300-054; Gibco) at 37°C and 5% CO_2_ for 3 min, transferred to phosphate-buffered saline (PBS), and then centrifuged at 300*g* for 5 min. The cells were then used for the respective experiments.

### Hypoxia treatment

Hypoxia preconditioning was performed by incubating Ad-MSCs for 30 min in the medium in a controlled atmosphere (anaerobic glove box; Plas Labs 855-AC) for free oxygen radical scavenging, with 95% N2 + 5% CO_2_ at 37 °C.

### Dox treatment

The cardiomyocytes were cultured with Dox to model cardiac injury. The concentration of doxorubicin was set as 0.5 μM and the exposure time as 24 h, as described previously [68].

### Isolation and characterization of exosomes

The exosomes were isolated and purified from the supernatants of MSCs and hypoxia-preconditioned MSCs, as described previously [69]. MSCs were cultured in exosome-depleted LG-DMEM. After the cells were cultured for 48 h, the supernatants were collected. The exosome quick extraction solution (total exosome isolation reagent from the cell culture medium) was added to the filtered solution at a 1:5 ratio and stored at 4°C for at least 12 h. The precipitation (exosomes) was dissolved in PBS and stored at −70°C. The size and concentration were determined by NTA. The morphology of exosomes was observed by TEM (JEM-1400plus). Western blot analysis was used for detecting exosomal markers CD63, CD81, Flotillin-1, and Tsg101. β-actin in the whole-cell lysate was used as control.

### Western blot analysis

The exosomes and cells were harvested, and total protein was extracted using RIPA solution. The protein samples were denatured, separated by 10% sodium dodecyl sulfate–polyacrylamide gel electrophoresis, and transferred to polyvinylidene difluoride membranes. The membranes were blocked with 5% fat-free milk for 2 h at room temperature and then incubated with CD63 (ab59479, 1:750), CD81 (ab79559, 1:500), Flotillin-1 (ab41927, 1:500), Tsg101 (ab125011, 1:500), p53 (ab131442, 1:750), p21 (ab109199, 1:750), ATG4a (CST,#7613, 1:500), and β-actin (ab179467, 1:1000) primary antibodies overnight at 4°C. The membranes were further incubated with IgG-horseradish peroxidase goat anti-rabbit/mouse secondary antibody (ab7090/ab97040: 1:2000) for 2 h at room temperature. The signals were developed by enhanced chemiluminescence (CST, #6883). The stained protein bands were visualized using a Bio-Rad ChemiDoc XRS imaging system and analyzed using Quantity One software.

### Cell cycle assay

Further, 70% cold anhydrous ethanol was used to fix the cells. Then, the cells were treated with propidium iodide (Sigma, St Louise, MO, USA) and RNase A. A flow cytometer equipped with Cell Quest software was used to detect the cell cycle distribution.

### Quantitative reverse transcription-polymerase chain reaction (qRT-PCR)

Real-time polymerase chain reaction (RT-PCR) was performed as described previously [70]. The primers are listed in Table 1. In brief, total RNA was extracted using TRIzol reagent; in all reactions, equal amounts of RNA were used (for miRNA transcription of 6 ng total RNA and for mRNA transcription 1000 ng total RNA), reverse transcribed to cDNA, and then amplified using an SYBR-Green master mix kit. Quantification cycle (Cq) cut-offs have been defined for miRNA (Cq 40) and mRNA (Cq 35) quantification. All procedures were performed in triplicate. The mRNA levels were calculated relative to the control GAPDH or U6 using the 2^-ΔΔCq^ method.

**Table 1:** Primer sequences.

**Genes**	**Sequences**
p53	F: 5' - CCGCAGTCAGATCCTAGCG -3'
	R: 5' - CCATTGCTTGGGACGGCAAGG -3'
p21	F: 5' - CAAGCTCTACCTTCCCACGG -3'
	R: 5' - GCCAGGGTATGTACATGAGG -3'
LncRNA-MALAT1	F: 5' - TGCGAGTTGTTCTCCGTCTA -3'
	R: 5' - TATCTGCGGTTTCCTCAAGC -3'
U6	F: 5'- GCTTCGGCAGCACATATACTAAAAT -3'
	R: 5'- CGCTTCACGAATTTGCGTGTCAT -3'
miR-92a-3p	F: 5' - TATTGCACTTGTCCCGGCCTGT -3'
	R: 5' - CCGAGGCGGCCGACATGTTT-3'
ATG4a	F: 5' - TGCTGGTTGGGGATGTATGC -3'
	R: 5' - GCGTTGGT ATTCTTTGGGTTGT-3'
Fabp3	F: 5' - CACCTGGAAGCTAGTGGACA -3'
	R: 5' - TTCCCTCCATCCAGTGTCAC -3'
Fabp4	F: 5' - CTGGTGGTGGAATGCGTCATGA -3'
	R: 5' - CAACGTCCCTTGGCTTATGCTCTCT -3'
Mtfp1	F: 5' - TAATCCACCCCATCGACAG -3'
	R: 5' - TCCACTGACGGGTACAGCTT-3'
Cox4i2	F: 5' - ATTTCCTCCAAAGCCGATCAC -3'
	R: 5' - GAGACAGCTGGGGATGCAAGTCA-3'
Hspa1a	F: 5' - GTGCTGACCAAGATGAAGGAG -3'
	R: 5' - GCTGCGAGTCGTTGAAGTAG -3'
Atp1b2	F: 5' - GAGGACGCACCAGTTTATGGG -3'
	R: 5' - GGGGTATGGTCGGAGACAGT -3'
GAPDH	F: 5'- TTGCCATCAATGACCCCTTCA -3'
	R: 5'- CGCCCCACTTGATTT TGGA -3'
siRNA-LncRNA-MALAT1	5'- TGCCTTTAGGATTCTAGACA -3'
siRNA-LncRNA-NT	5'- CCTTCCCTGAAGGTTCCTCC -3'
siRNA-ATG4a	5'- AGGACCTGCGCTTCCAGA -3'
siRNA-NT	5'- AGCGTGCGGCTTCTGAAG -3'

### Senescence-associated β-galactosidase assay

The cellular senescence was measured using senescence-associated β-galactosidase (SA-β-gal) assay (Cell Signaling Technology, MA, USA). Briefly, the cells at the density of 2 × 10^4^ were washed with PBS, fixed with 2% paraformaldehyde for 30 min at room temperature, and incubated with a fresh SA-β-gal staining solution as previously described [14]. The results were presented as a ratio of the SA-β-gal-positive cells (cytoplasm blue stained) to the total cells for at least 100 cells per treatment per experiment.

### Microarray analysis

The exosomes and cardiomyocytes from triplicate groups were lysed immediately in 500 μL of TRIzol (ThermoFisher Scientific, MA, USA) and stored at −80°C before purification using a standard phenol–chloroform extraction protocol with an RNAqueous Micro Kit (ThermoFisher Scientific). RNA was used for miRNA microarray analysis (miRCURY LNA microRNA Array, Exiqon A/S, Vedbaek, Denmark), according to the manufacturer’s recommendations.

### Small interfering RNA transfection

LncRNA-MALAT1 expression in MSCs was knocked down using small interfering (si)RNAs, with a nontargeting siRNA as a NC (Invitrogen). ATG4a expression in cardiomyocytes was also knocked down using siRNAs. The procedures were conducted, as described previously [17]. The target sequences are listed in Table 1. The transfection efficiency was detected using qRT-PCR and Western blot analysis.

### MiR-92a-3p overexpression

The cardiomyocytes were seeded into six-well plates at a density of 1 × 10^5^ cells per well and incubated for 12 h. To induce the overexpression of miR-92-3p, the cells were transfected with miR-92-3p mimic or NC mimic (Pre-miR miRNA Precursors, Life Technologies, Karlsruhe, Germany) using X-treme transfection reagent (Roche Applied Science, Penzberg, Germany), according to the manufacturer’s protocol. The cells were harvested for further analysis 48 h after transfection, and the transfection efficiency was analyzed using qRT-PCR.

### Luciferase reporter assay

The 3′-untranslated regions (UTRs) of lncRNA-MALAT1 and ATG4a were synthesized, annealed, and inserted into the *Sac*I and *Hind*III sites of the pmiR-reporter luciferase vector (Ambion), downstream of the luciferase stop codon to induce the mutagenesis of lncRNA-MALAT1 and ATG4a. The constructs were validated by sequencing. The cardiomyocytes were seeded into a 24-well plate for luciferase assay. After overnight culture, the cells were co-transfected with a WT or mutated plasmid and equal amounts of miR-92a-3p mimic or miR-NC mimic. Luciferase assays were performed using a dual-luciferase reporter assay system (Promega) 24 h after transfection.

### Statistical analysis

Data were expressed as the mean ± standard deviation (SD). Differences between groups were tested by one-way analysis of variance, and comparisons between two groups were evaluated using the Student *t* test. Analyses were performed using SPSS package v19.0 (SPSS Inc., IL, USA). A *P* value less than 0.05 was considered statistically significant.

## References

[1] Ghigo A, Li M, Hirsch E. New signal transduction paradigms in anthracycline-induced cardiotoxicity. Biochim Biophys Acta. 2016 (7 Pt B); 1863:1916–25. 10.1016/j.bbamcr.2016.01.021 26828775

[2] Wouters KA, Kremer LC, Miller TL, Herman EH, Lipshultz SE. Protecting against anthracycline-induced myocardial damage: a review of the most promising strategies. Br J Haematol. 2005; 131:561–78. 10.1111/j.1365-2141.2005.05759.x 16351632

[3] Xinyong C, Zhiyi Z, Lang H, Peng Y, Xiaocheng W, Ping Z, Liang S. The role of toll-like receptors in myocardial toxicity induced by doxorubicin. Immunol Lett. 2020; 217:56–64. 10.1016/j.imlet.2019.11.001 31707054

[4] Gilliam LA, Moylan JS, Patterson EW, Smith JD, Wilson AS, Rabbani Z, Reid MB. Doxorubicin acts via mitochondrial ROS to stimulate catabolism in C2C12 myotubes. Am J Physiol Cell Physiol. 2012; 302:C195–202. 10.1152/ajpcell.00217.2011 21940668 PMC3328915

[5] Zhang S, Liu X, Bawa-Khalfe T, Lu LS, Lyu YL, Liu LF, Yeh ET. Identification of the molecular basis of doxorubicin-induced cardiotoxicity. Nat Med. 2012; 18:1639–42. 10.1038/nm.2919 23104132

[6] Natsumeda M, Florea V, Rieger AC, Tompkins BA, Banerjee MN, Golpanian S, Fritsch J, Landin AM, Kashikar ND, Karantalis V, Loescher VY, Hatzistergos KE, Bagno L, et al. A Combination of Allogeneic Stem Cells Promotes Cardiac Regeneration. J Am Coll Cardiol. 2017; 70:2504–15. 10.1016/j.jacc.2017.09.036 29145950 PMC5796680

[7] Deuse T, Peter C, Fedak PW, Doyle T, Reichenspurner H, Zimmermann WH, Eschenhagen T, Stein W, Wu JC, Robbins RC, Schrepfer S. Hepatocyte growth factor or vascular endothelial growth factor gene transfer maximizes mesenchymal stem cell-based myocardial salvage after acute myocardial infarction. Circulation. 2009 (Suppl); 120:S247–54. 10.1161/CIRCULATIONAHA.108.843680 19752375

[8] Kim HY, Kumar H, Jo MJ, Kim J, Yoon JK, Lee JR, Kang M, Choo YW, Song SY, Kwon SP, Hyeon T, Han IB, Kim BS. Therapeutic Efficacy-Potentiated and Diseased Organ-Targeting Nanovesicles Derived from Mesenchymal Stem Cells for Spinal Cord Injury Treatment. Nano Lett. 2018; 18:4965–75. 10.1021/acs.nanolett.8b01816 29995418

[9] Jella KK, Nasti TH, Li Z, Malla SR, Buchwald ZS, Khan MK. Exosomes, Their Biogenesis and Role in Inter-Cellular Communication, Tumor Microenvironment and Cancer Immunotherapy. Vaccines (Basel). 2018; 6:E69. 10.3390/vaccines6040069 30261592 PMC6313856

[10] Zhang Y, Liu Y, Liu H, Tang WH. Exosomes: biogenesis, biologic function and clinical potential. Cell Biosci. 2019; 9:19. 10.1186/s13578-019-0282-2 30815248 PMC6377728

[11] Singla DK, Johnson TA, Tavakoli Dargani Z. Exosome Treatment Enhances Anti-Inflammatory M2 Macrophages and Reduces Inflammation-Induced Pyroptosis in Doxorubicin-Induced Cardiomyopathy. Cells. 2019; 8:E1224. 10.3390/cells8101224 31600901 PMC6830113

[12] Egger D, Schwedhelm I, Hansmann J, Kasper C. Hypoxic Three-Dimensional Scaffold-Free Aggregate Cultivation of Mesenchymal Stem Cells in a Stirred Tank Reactor. Bioengineering (Basel). 2017; 4:E47. 10.3390/bioengineering4020047 28952526 PMC5590473

[13] Kinnaird T, Stabile E, Burnett MS, Lee CW, Barr S, Fuchs S, Epstein SE. Marrow-derived stromal cells express genes encoding a broad spectrum of arteriogenic cytokines and promote in vitro and in vivo arteriogenesis through paracrine mechanisms. Circ Res. 2004; 94:678–85. 10.1161/01.RES.0000118601.37875.AC 14739163

[14] Hu Y, Xia W, Hou M. Macrophage migration inhibitory factor serves a pivotal role in the regulation of radiation-induced cardiac senescencethrough rebalancing the microRNA-34a/sirtuin 1 signaling pathway. Int J Mol Med. 2018; 42:2849–58. 10.3892/ijmm.2018.3838 30226567

[15] Almeria C, Weiss R, Roy M, Tripisciano C, Kasper C, Weber V, Egger D. Hypoxia Conditioned Mesenchymal Stem Cell-Derived Extracellular Vesicles Induce Increased Vascular Tube Formation *in vitro.* Front Bioeng Biotechnol. 2019; 7:292. 10.3389/fbioe.2019.00292 31709251 PMC6819375

[16] Ponnusamy M, Liu F, Zhang YH, Li RB, Zhai M, Liu F, Zhou LY, Liu CY, Yan KW, Dong YH, Wang M, Qian LL, Shan C, et al. Long Noncoding RNA CPR (Cardiomyocyte Proliferation Regulator) Regulates Cardiomyocyte Proliferation and Cardiac Repair. Circulation. 2019; 139:2668–84. 10.1161/CIRCULATIONAHA.118.035832 30832495

[17] Xie Z, Xia W, Hou M. Long intergenic non-coding RNA-p21 mediates cardiac senescence via the Wnt/β-catenin signaling pathway in doxorubicin-induced cardiotoxicity. Mol Med Rep. 2018; 17:2695–704. 10.3892/mmr.2017.8169 29207090

[18] Gutschner T, Hämmerle M, Diederichs S. MALAT1— a paradigm for long noncoding RNA function in cancer. J Mol Med (Berl). 2013; 91:791–801. 10.1007/s00109-013-1028-y 23529762

[19] Stone JK, Kim JH, Vukadin L, Richard A, Giannini HK, Lim SS, Tan M, Ahn EE. Hypoxia induces cancer cell-specific chromatin interactions and increases MALAT1 expression in breast cancer cells. J Biol Chem. 2019; 294:11213–24. 10.1074/jbc.RA118.006889 31167784 PMC6643033

[20] Yang X, Yang J, Lei P, Wen T. LncRNA MALAT1 shuttled by bone marrow-derived mesenchymal stem cells-secreted exosomes alleviates osteoporosis through mediating microRNA-34c/SATB2 axis. Aging (Albany NY). 2019; 11:8777–91. 10.18632/aging.102264 31659145 PMC6834402

[21] Qiao L, Hu S, Liu S, Zhang H, Ma H, Huang K, Li Z, Su T, Vandergriff A, Tang J, Allen T, Dinh PU, Cores J, et al. microRNA-21-5p dysregulation in exosomes derived from heart failure patients impairs regenerative potential. J Clin Invest. 2019; 129:2237–50. 10.1172/JCI123135 31033484 PMC6546482

[22] Rezaie J, Rahbarghazi R, Pezeshki M, Mazhar M, Yekani F, Khaksar M, Shokrollahi E, Amini H, Hashemzadeh S, Sokullu SE, Tokac M. Cardioprotective role of extracellular vesicles: A highlight on exosome beneficial effects in cardiovascular diseases. J Cell Physiol. 2019; 234:21732–45. 10.1002/jcp.28894 31140622

[23] Rogg EM, Abplanalp WT, Bischof C, John D, Schulz MH, Krishnan J, Fischer A, Poluzzi C, Schaefer L, Bonauer A, Zeiher AM, Dimmeler S. Analysis of Cell Type-Specific Effects of MicroRNA-92a Provides Novel Insights Into Target Regulation and Mechanism of Action. Circulation. 2018; 138:2545–58. 10.1161/CIRCULATIONAHA.118.034598 30571345

[24] Singh KK, Yanagawa B, Quan A, Wang R, Garg A, Khan R, Pan Y, Wheatcroft MD, Lovren F, Teoh H, Verma S. Autophagy gene fingerprint in human ischemia and reperfusion. J Thorac Cardiovasc Surg. 2014; 147:1065–1072.e1. 10.1016/j.jtcvs.2013.04.042 23778083

[25] Sahin E, Colla S, Liesa M, Moslehi J, Müller FL, Guo M, Cooper M, Kotton D, Fabian AJ, Walkey C, Maser RS, Tonon G, Foerster F, et al. Telomere dysfunction induces metabolic and mitochondrial compromise. Nature. 2011; 470:359–65. 10.1038/nature09787 21307849 PMC3741661

[26] Cappetta D, De Angelis A, Sapio L, Prezioso L, Illiano M, Quaini F, Rossi F, Berrino L, Naviglio S, Urbanek K. Oxidative Stress and Cellular Response to Doxorubicin: A Common Factor in the Complex Milieu of Anthracycline Cardiotoxicity. Oxid Med Cell Longev. 2017; 2017:1521020. 10.1155/2017/1521020 29181122 PMC5664340

[27] Chua S, Lee FY, Chiang HJ, Chen KH, Lu HI, Chen YT, Yang CC, Lin KC, Chen YL, Kao GS, Chen CH, Chang HW, Yip HK. The cardioprotective effect of melatonin and exendin-4 treatment in a rat model of cardiorenal syndrome. J Pineal Res. 2016; 61:438–56. 10.1111/jpi.12357 27465663

[28] Rebbaa A, Zheng X, Chou PM, Mirkin BL. Caspase inhibition switches doxorubicin-induced apoptosis to senescence. Oncogene. 2003; 22:2805–11. 10.1038/sj.onc.1206366 12743603

[29] Childs BG, Durik M, Baker DJ, van Deursen JM. Cellular senescence in aging and age-related disease: from mechanisms to therapy. Nat Med. 2015; 21:1424–35. 10.1038/nm.4000 26646499 PMC4748967

[30] Finkelman BS, Putt M, Wang T, Wang L, Narayan H, Domchek S, DeMichele A, Fox K, Matro J, Shah P, Clark A, Bradbury A, Narayan V, et al. Arginine-Nitric Oxide Metabolites and Cardiac Dysfunction in Patients With Breast Cancer. J Am Coll Cardiol. 2017; 70:152–62. 10.1016/j.jacc.2017.05.019 28683962 PMC5665653

[31] Zilinyi R, Czompa A, Czegledi A, Gajtko A, Pituk D, Lekli I, Tosaki A. The Cardioprotective Effect of Metformin in Doxorubicin-Induced Cardiotoxicity: The Role of Autophagy. Molecules. 2018; 23:E1184. 10.3390/molecules23051184 29762537 PMC6100061

[32] Li DL, Wang ZV, Ding G, Tan W, Luo X, Criollo A, Xie M, Jiang N, May H, Kyrychenko V, Schneider JW, Gillette TG, Hill JA. Doxorubicin Blocks Cardiomyocyte Autophagic Flux by Inhibiting Lysosome Acidification. Circulation. 2016; 133:1668–87. 10.1161/CIRCULATIONAHA.115.017443 26984939 PMC4856587

[33] Sun R, Li X, Liu M, Zeng Y, Chen S, Zhang P. Advances in stem cell therapy for cardiovascular disease (Review). Int J Mol Med. 2016; 38:23–29. Review 10.3892/ijmm.2016.2607 27220939 PMC4899023

[34] Faiella W, Atoui R. Therapeutic use of stem cells for cardiovascular disease. Clin Transl Med. 2016; 5:34. 10.1186/s40169-016-0116-3 27539581 PMC4990528

[35] Garbade J, Dhein S, Lipinski C, Aupperle H, Arsalan M, Borger MA, Barten MJ, Lehmann S, Walther T, Mohr FW. Bone marrow-derived stem cells attenuate impaired contractility and enhance capillary density in a rabbit model of Doxorubicin-induced failing hearts. J Card Surg. 2009; 24:591–99. 10.1111/j.1540-8191.2009.00844.x 19538224

[36] Abushouk AI, Salem AM, Saad A, Afifi AM, Afify AY, Afify H, Salem HS, Ghanem E, Abdel-Daim MM. Mesenchymal Stem Cell Therapy for Doxorubicin-Induced Cardiomyopathy: Potential Mechanisms, Governing Factors, and Implications of the Heart Stem Cell Debate. Front Pharmacol. 2019; 10:635. 10.3389/fphar.2019.00635 31258475 PMC6586740

[37] Hao D, He C, Ma B, Lankford L, Reynaga L, Farmer DL, Guo F, Wang A. Hypoxic Preconditioning Enhances Survival and Proangiogenic Capacity of Human First Trimester Chorionic Villus-Derived Mesenchymal Stem Cells for Fetal Tissue Engineering. Stem Cells Int. 2019; 2019:9695239. 10.1155/2019/9695239 31781252 PMC6874947

[38] Hentze H, Soong PL, Wang ST, Phillips BW, Putti TC, Dunn NR. Teratoma formation by human embryonic stem cells: evaluation of essential parameters for future safety studies. Stem Cell Res. 2009; 2:198–210. 10.1016/j.scr.2009.02.002 19393593

[39] Gonzalez-King H, García NA, Ontoria-Oviedo I, Ciria M, Montero JA, Sepúlveda P. Hypoxia Inducible Factor-1α Potentiates Jagged 1-Mediated Angiogenesis by Mesenchymal Stem Cell-Derived Exosomes. Stem Cells. 2017; 35:1747–59. 10.1002/stem.2618 28376567

[40] Singla DK. Stem cells and exosomes in cardiac repair. Curr Opin Pharmacol. 2016; 27:19–23. 10.1016/j.coph.2016.01.003 26848944

[41] Gnecchi M, He H, Noiseux N, Liang OD, Zhang L, Morello F, Mu H, Melo LG, Pratt RE, Ingwall JS, Dzau VJ. Evidence supporting paracrine hypothesis for Akt-modified mesenchymal stem cell-mediated cardiac protection and functional improvement. FASEB J. 2006; 20:661–69. 10.1096/fj.05-5211com 16581974

[42] Rosová I, Dao M, Capoccia B, Link D, Nolta JA. Hypoxic preconditioning results in increased motility and improved therapeutic potential of human mesenchymal stem cells. Stem Cells. 2008; 26:2173–82. 10.1634/stemcells.2007-1104 18511601 PMC3017477

[43] Mittelbrunn M, Sánchez-Madrid F. Intercellular communication: diverse structures for exchange of genetic information. Nat Rev Mol Cell Biol. 2012; 13:328–35. 10.1038/nrm3335 22510790 PMC3738855

[44] Sun Q, Hao Q, Prasanth KV. Nuclear Long Noncoding RNAs: Key Regulators of Gene Expression. Trends Genet. 2018; 34:142–57. 10.1016/j.tig.2017.11.005 29249332 PMC6002860

[45] Wang K, Liu CY, Zhou LY, Wang JX, Wang M, Zhao B, Zhao WK, Xu SJ, Fan LH, Zhang XJ, Feng C, Wang CQ, Zhao YF, Li PF. APF lncRNA regulates autophagy and myocardial infarction by targeting miR-188-3p. Nat Commun. 2015; 6:6779. 10.1038/ncomms7779 25858075

[46] Cai B, Ma W, Ding F, Zhang L, Huang Q, Wang X, Hua B, Xu J, Li J, Bi C, Guo S, Yang F, Han Z, et al. The Long Noncoding RNA CAREL Controls Cardiac Regeneration. J Am Coll Cardiol. 2018; 72:534–50. 10.1016/j.jacc.2018.04.085 30056829

[47] Du WW, Yang W, Chen Y, Wu ZK, Foster FS, Yang Z, Li X, Yang BB. Foxo3 circular RNA promotes cardiac senescence by modulating multiple factors associated with stress and senescence responses. Eur Heart J. 2017; 38:1402–12. 10.1093/eurheartj/ehw001 26873092

[48] Tripathi V, Shen Z, Chakraborty A, Giri S, Freier SM, Wu X, Zhang Y, Gorospe M, Prasanth SG, Lal A, Prasanth KV. Long noncoding RNA MALAT1 controls cell cycle progression by regulating the expression of oncogenic transcription factor B-MYB. PLoS Genet. 2013; 9:e1003368. 10.1371/journal.pgen.1003368 23555285 PMC3605280

[49] Liu JY, Yao J, Li XM, Song YC, Wang XQ, Li YJ, Yan B, Jiang Q. Pathogenic role of lncRNA-MALAT1 in endothelial cell dysfunction in diabetes mellitus. Cell Death Dis. 2014; 5:e1506. 10.1038/cddis.2014.466 25356875 PMC4649539

[50] Wang K, Sun T, Li N, Wang Y, Wang JX, Zhou LY, Long B, Liu CY, Liu F, Li PF. MDRL lncRNA regulates the processing of miR-484 primary transcript by targeting miR-361. PLoS Genet. 2014; 10:e1004467. 10.1371/journal.pgen.1004467 25057983 PMC4109843

[51] Wang JX, Zhang XJ, Li Q, Wang K, Wang Y, Jiao JQ, Feng C, Teng S, Zhou LY, Gong Y, Zhou ZX, Liu J, Wang JL, Li PF. MicroRNA-103/107 Regulate Programmed Necrosis and Myocardial Ischemia/Reperfusion Injury Through Targeting FADD. Circ Res. 2015; 117:352–63. 10.1161/CIRCRESAHA.117.305781 26038570

[52] Hinkel R, Penzkofer D, Zühlke S, Fischer A, Husada W, Xu QF, Baloch E, van Rooij E, Zeiher AM, Kupatt C, Dimmeler S. Inhibition of microRNA-92a protects against ischemia/reperfusion injury in a large-animal model. Circulation. 2013; 128:1066–75. 10.1161/CIRCULATIONAHA.113.001904 23897866

[53] Mizushima N, Yoshimori T, Levine B. Methods in mammalian autophagy research. Cell. 2010; 140:313–26. 10.1016/j.cell.2010.01.028 20144757 PMC2852113

[54] Preiss R, Tyrawa C, van der Merwe G. Autophagy gene overexpression in Saccharomyces cerevisiae perturbs subcellular organellar function and accumulates ROS to accelerate cell death with relevance to sparkling wine production. Appl Microbiol Biotechnol. 2018; 102:8447–64. 10.1007/s00253-018-9304-y 30120525

[55] Pan JA, Tang Y, Yu JY, Zhang H, Zhang JF, Wang CQ, Gu J. miR-146a attenuates apoptosis and modulates autophagy by targeting TAF9b/P53 pathway in doxorubicin-induced cardiotoxicity. Cell Death Dis. 2019; 10:668. 10.1038/s41419-019-1901-x 31511497 PMC6739392

[56] Ichikawa Y, Ghanefar M, Bayeva M, Wu R, Khechaduri A, Naga Prasad SV, Mutharasan RK, Naik TJ, Ardehali H. Cardiotoxicity of doxorubicin is mediated through mitochondrial iron accumulation. J Clin Invest. 2014; 124:617–30. 10.1172/JCI72931 24382354 PMC3904631

[57] Baker DJ, Peleg S. Biphasic Modeling of Mitochondrial Metabolism Dysregulation during Aging. Trends Biochem Sci. 2017; 42:702–11. 10.1016/j.tibs.2017.06.005 28669456

[58] Goldberg IJ, Trent CM, Schulze PC. Lipid metabolism and toxicity in the heart. Cell Metab. 2012; 15:805–12. 10.1016/j.cmet.2012.04.006 22682221 PMC3387529

[59] Octavia Y, Tocchetti CG, Gabrielson KL, Janssens S, Crijns HJ, Moens AL. Doxorubicin-induced cardiomyopathy: from molecular mechanisms to therapeutic strategies. J Mol Cell Cardiol. 2012; 52:1213–25. 10.1016/j.yjmcc.2012.03.006 22465037

[60] Lenneman CG, Sawyer DB. Cardio-Oncology: An Update on Cardiotoxicity of Cancer-Related Treatment. Circ Res. 2016; 118:1008–20. 10.1161/CIRCRESAHA.115.303633 26987914

[61] Spitalny GL, Havell EA. Monoclonal antibody to murine gamma interferon inhibits lymphokine-induced antiviral and macrophage tumoricidal activities. J Exp Med. 1984; 159:1560–65. 10.1084/jem.159.5.1560 6425450 PMC2187308

[62] Zhuang L, Li C, Chen Q, Jin Q, Wu L, Lu L, Yan X, Chen K. Fatty acid-binding protein 3 contributes to ischemic heart injury by regulating cardiac myocyte apoptosis and MAPK pathways. Am J Physiol Heart Circ Physiol. 2019; 316:H971–84. 10.1152/ajpheart.00360.2018 30735072

[63] Peri-Okonny P, Baskin KK, Iwamoto G, Mitchell JH, Smith SA, Kim HK, Szweda LI, Bassel-Duby R, Fujikawa T, Castorena CM, Richardson J, Shelton JM, Ayers C, et al. High-Phosphate Diet Induces Exercise Intolerance and Impairs Fatty Acid Metabolism in Mice. Circulation. 2019; 139:1422–34. 10.1161/CIRCULATIONAHA.118.037550 30612451 PMC6411426

[64] Aung LH, Li R, Prabhakar BS, Li P. Knockdown of Mtfp1 can minimize doxorubicin cardiotoxicity by inhibiting Dnm1l-mediated mitochondrial fission. J Cell Mol Med. 2017; 21:3394–404. 10.1111/jcmm.13250 28643438 PMC5706585

[65] Zhong W, Zhu H, Sheng F, Tian Y, Zhou J, Chen Y, Li S, Lin J. Activation of the MAPK11/12/13/14 (p38 MAPK) pathway regulates the transcription of autophagy genes in response to oxidative stress induced by a novel copper complex in HeLa cells. Autophagy. 2014; 10:1285–300. 10.4161/auto.28789 24905917 PMC4203553

[66] Grossman LI, Purandare N, Arshad R, Gladyck S, Somayajulu M, Hüttemann M, Aras S. MNRR1, a Biorganellar Regulator of Mitochondria. Oxid Med Cell Longev. 2017; 2017:6739236. 10.1155/2017/6739236 28685009 PMC5480048

[67] Figtree GA, Liu CC, Bibert S, Hamilton EJ, Garcia A, White CN, Chia KK, Cornelius F, Geering K, Rasmussen HH. Reversible oxidative modification: a key mechanism of Na+-K+ pump regulation. Circ Res. 2009; 105:185–93. 10.1161/CIRCRESAHA.109.199547 19542013

[68] Xia W, Hou M. Mesenchymal stem cells confer resistance to doxorubicin-induced cardiac senescence by inhibiting microRNA-34a. Oncol Lett. 2018; 15:10037–46. 10.3892/ol.2018.8438 29928373 PMC6004710

[69] Li T, Yan Y, Wang B, Qian H, Zhang X, Shen L, Wang M, Zhou Y, Zhu W, Li W, Xu W. Exosomes derived from human umbilical cord mesenchymal stem cells alleviate liver fibrosis. Stem Cells Dev. 2013; 22:845–54. 10.1089/scd.2012.0395 23002959 PMC3585469

[70] Shan G, Tang T, Xia Y, Qian HJ. Long non-coding RNA NEAT1 promotes bladder progression through regulating miR-410 mediated HMGB1. Biomed Pharmacother. 2020; 121:109248. 10.1016/j.biopha.2019.109248 31734579

